# 

**Published:** 1906-06-30

**Authors:** 


					I H THE HOSPITAL. 111 '1 June 30th 1906.
t WKi I f I V,.; : hf i; '.S rr?" lUm\ II?!fl
JUL 9- fjjrr:d
P I T A L-
^0-1,032. Vol. XL. ES5S1S Saturday,
June 30th, 1906.
[Sabfeelf^TermVr^>?raWEPENG^

				

